# Effect of Integrated Yoga as an Adjuvant to Standard Care for Panic Disorder: A Randomized Control Trial Study

**DOI:** 10.7759/cureus.53286

**Published:** 2024-01-31

**Authors:** Vishwa Sree Yadla, Patil NJ, Prabhakar Kamarthy, Mohan Reddy Matti

**Affiliations:** 1 Department of Integrative Medicine, Sri Devaraj Urs Academy of Higher Education and Research, Kolar, IND; 2 Department of Yoga, Center for Integrative Medicine and Research (CIMR), Manipal, IND; 3 Department of General Medicine, Sri Devaraj Urs Medical College, Sri Devaraj Urs Academy of Higher Education and Research, Kolar, IND; 4 Department of Psychiatry, Sri Devaraj Urs Medical College, Sri Devaraj Urs Academy of Higher Education and Research, Kolar, IND

**Keywords:** well-being, mindfulness, mental health, complementary therapies, holistic intervention, who quality of life – bref, hamilton-anxiety rating scale, panic disorder, yoga

## Abstract

Background: Individuals wrestling with panic disorder (PD) know all too well its debilitating impact. Sudden, intense fear episodes disrupt lives and erode well-being. Fortunately, integrating complementary therapies like yoga with standard treatment offers a glimmer of hope for improved outcomes. Yoga's unique blend of physical postures (asanas), breathing exercises (pranayama), and meditative practices holds promise for mitigating anxiety and fostering a sense of inner peace, potentially making it a valuable tool in the fight against panic disorder.

Methods and materials: This study investigated the effect of yoga as an adjuvant to standard care for panic disorder. Sixty-four panic disorder patients of both genders previously diagnosed with panic disorder according to the Diagnostic and Statistical Manual of Mental Disorders (DSM-5) criteria were randomly assigned to the yoga group (n = 32) and the control group. The yoga group participated in integrated yoga sessions lasting 60 minutes, five days a week, for 12 weeks. Both groups received standard care. Pre- and post-intervention data were collected for HAM-A and WHOQOL-BREF.

Results: The yoga group exhibited a significant reduction in HAM-A scores (Pre: 49.13 ± 4.55, Post: 13.53 ± 5.54, p < 0.001) with a substantial effect size of 7.02. Quality of life significantly improved across all domains (physical, psychological, social, and environmental) in the yoga group (p < 0.001), demonstrating effect sizes ranging from 4.11 to 4.57. Control group participants also experienced improvements, though less pronounced. Between-group comparisons revealed significant differences in anxiety reduction (p = 0.042) and quality of life enhancement (p < 0.001), favouring the yoga group.

Conclusion: The results suggest that yoga can be a valuable complementary or alternative approach to traditional treatments for anxiety disorders.

## Introduction

Panic disorder (PD) poses a significant challenge to mental health professionals due to its debilitating impact on individuals' daily lives and overall well-being. Characterized by recurrent and unexpected panic attacks, PD often manifests alongside heightened anxiety, physiological distress, and persistent apprehension about future attacks [1]. During a panic attack, individuals experience a variety of physical symptoms, such as a rapid heartbeat, hyperventilation (rapid breathing), perspiration (excessive sweating), dizziness, dyspnea (shortness of breath), trembling, and an overwhelming sense of fear [2-4]. These physical symptoms can be distressing and can lead to a fear of losing control or going crazy, as well as a fear of dying. The multifaceted nature of PD necessitates comprehensive treatment approaches to address its complex etiology and mitigate the associated symptoms effectively.

Current treatments for panic disorder include psychotherapeutic and pharmacological interventions, both supported by a great amount of empirical evidence [5,6]. Psychotherapeutic interventions refer to therapies that involve talking and counselling, such as cognitive-behavioural therapy (CBT), which focuses on identifying and changing negative thought patterns and behaviours [7,8]. Pharmacological interventions refer to the use of medications, such as selective serotonin reuptake inhibitors (SSRIs), which help regulate brain chemicals involved in mood and anxiety [9,10]. These treatments have been extensively studied and have been effective in reducing the symptoms of panic disorder. However, studies suggest that many individuals do not seek professional help, which indicates the need for reliable and appropriate self-help strategies [11]. This may be due to various reasons, such as stigma, lack of access to mental health services, or personal preferences. As a result, there is a need for self-help strategies that individuals can use on their own to manage their symptoms and improve their well-being. Many individuals with panic disorder express dissatisfaction with relying solely on medication to manage their symptoms for their entire lives. While medication can be helpful, it may not address the underlying causes of panic disorder or provide long-term solutions. Traditional psychotherapy, although effective, can also be costly and time-consuming, especially if it needs to be continued for years. However, therapies like CBT are known to be relatively brief and effective in treating panic disorder, making them a more accessible option for many individuals. Clinical trials have shown that anxiolytic drugs have limited long-term efficacy, can cause dependency, sleepiness, sexual dysfunction, and affect cognition and memory.

A national survey in the United States found that approximately two-thirds of the sample utilized complementary therapies for mental disorders, such as acupuncture, meditation, and yoga, with positive results. These complementary therapies include mind-body interventions, such as meditation and yoga. These practices are believed to have positive effects on mental health and well-being. Yoga aims to achieve a unified state of consciousness and self-realization. It involves techniques such as physical postures, controlled breathing, relaxation, and meditation. Research has shown that yoga and meditation have significant psycho-physiological benefits, including improvements in emotional self-regulation, reductions in depression, stress, and anxiety levels, and improvements in mood, quality of life, and well-being. Yoga is also being used as a therapeutic intervention in the treatment of mental disorders.

This study seeks to explore the potential benefits of integrating yoga as an adjuvant to standard care for individuals diagnosed with PD. The rationale behind incorporating yoga lies in its ability to address not only the physiological symptoms but also the psycho-social and emotional aspects associated with PD. The integration of yoga into treatment plans offers a holistic approach that considers the interconnectedness of mind and body, aiming for a more comprehensive therapeutic impact.

## Materials and methods

Ethical considerations

The study is approved by the Ethics Committee, Ref. No. SDUAHER/KLR/CEC/67/2021-22. It was registered in the Clinical Trials Registry of India (CTRI No. - CTRI/2022/04/041907). Written informed consent was obtained from all the participants before the study, as it was voluntary participation.

Methods

This study employs a randomized controlled trial (RCT) design to rigorously investigate the impact of integrated yoga as an adjuvant to standard care. We recruited individuals diagnosed with PD from a tertiary care teaching hospital in the Kolar district of Karnataka state, India, for a period of October 13, 2022, to October 13, 2023. The RCT design is chosen for its ability to minimize selection bias, allowing for a robust comparison between the intervention group receiving integrated yoga and the control group receiving standard care alone. They were randomly divided into two groups: control (n = 32; age: 25.69 ± 6.27 years) and yoga (n = 32; age: 25.84 ± 5.84 years) using a random number generator (www.randomizer.org) [12]. The assessment of HAM-A and QOL was carried out at two baseline points and 12 weeks after the intervention.

Participants

Sixty-four panic disorder patients of both genders previously diagnosed with panic disorder according to Diagnostic and Statistical Manual of Mental Disorders (DSM-5) criteria who met the inclusion criteria between the age groups of 18 and 35, Willing to participate in both standard care and the assigned intervention (yoga or control), and medically stable with no contraindications to yoga or the chosen medication for PD. The exclusion criteria included a history of other psychiatric disorders or physical conditions that would affect their ability to perform yoga. Base assessments were conducted and randomly assigned to the yoga group (n = 32) and the control group (n = 32). Both groups were monitored with standard care.

Control Group

Participants in the control group receive standard care for panic disorder, consistent with prevailing treatment guidelines. Standard care may include pharmacotherapy and cognitive-behavioural therapy. The control group serves as a comparator to isolate the specific effects of the yoga intervention.

Yoga Group

**Figure 1:**
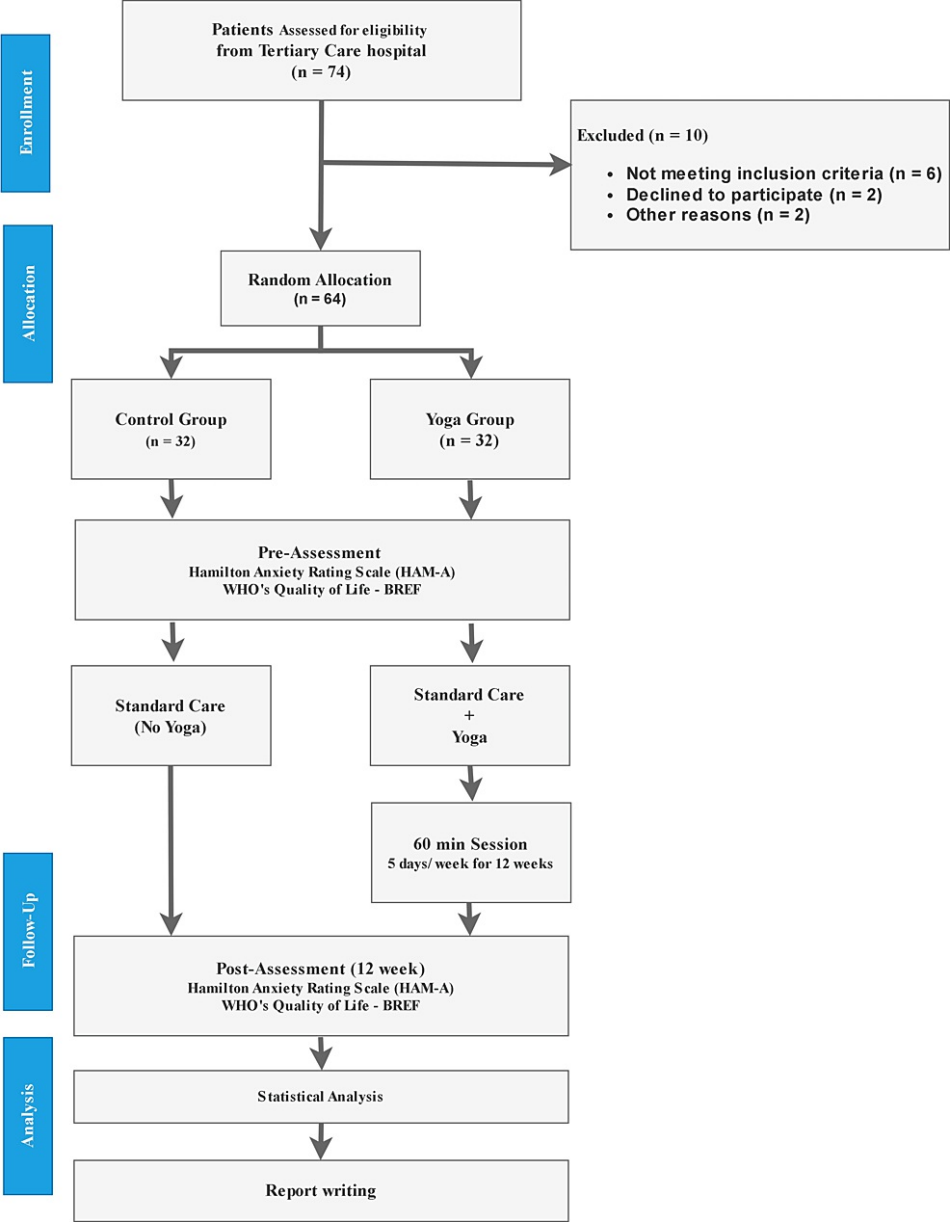
Flow diagram of the study

Participants in the yoga group underwent a 12-week intervention consisting of 60 minutes of yoga, five times a week. The integrated yoga sessions have been designed to combine the techniques of loosening exercise (sookshma vyayama), sunsalutations (suryanamaskara), physical postures (asanas), breathing practices (pranayama), and anapana meditation, as listed in Table 1, to address both the physical and psychological aspects of PD. Qualified yoga instructors with expertise in mental health guide the sessions, ensuring standardized delivery of the intervention. The integrated yoga module designed for individuals with panic disorder has been meticulously reviewed and approved by three certified yoga experts. No dropouts were observed in the study. Figure 1 shows the flow diagram of the study profile.

**Table 1:** Yoga module for panic disorder

Sl. No.	Name of the practice		Duration (minutes)
1.	Starting prayer	Patanjali prayer	1
2.	Yogic micro exercises and gross exercises (yogic sukshma and sthula vyayama)	Intelligence development process (Medha shakti vikasaka kriya) memory power development process (Smarana shakti vikasaka kriya) Intelligence and cognitive development process (Buddhi tatha dhriti shakti vikasaka kriya) upward movement (Urdhwagati)	5
3.	Sun salutation (suryanamaskar)		5
4.	Yogasana	Standing posture, mountain pose (Tadasana)	2
		Standing spinal twist pose (Katichakrasana)	2
		Supine posture wind-relieving pose (Pavanamuktasana)	2
		*Bridge pose (*Sethubhandasana)	2
		Shoulder stand pose (Sarvangasana)	2
		Easy fish pose (Sarala Matsyasana)	2
		Prone posture crocodile pose (Makarasana)	2
		Bow pose (Dhanurasana)	2
		Sitting posture cow face pose (Gomukhasana)	2
		Spinal twist pose (Vakrasana)	2
		Corpse pose (Shavasana)	5
5.	Breathing practice (Pranayama)	Bellows breath (Bhastrika)	1
		Alternate nostril breathing (Nadishuddi)	5
		Cooling breath (Shitali)	2
		Humming bee breath (Bhramari)	5
6.	Meditation	Observing natural, normal respiration (Anapana)	10
7.	Closing prayer	Sarve bhavantu sukinaha	1
		Total	60

Data collection/assessment

Demographic details like age, gender, education, qualification, locality, marital status, and occupation were collected using a structured questionnaire. Data were collected on the same day of recruitment and the last day of the 12th week. The assessment of anxiety was conducted using the 14-item Hamilton Anxiety Rating Scale, which is a widely used and well-established tool for measuring the severity of anxiety symptoms covering psychological, somatic, and autonomic domains. Additionally, the quality of life was assessed using the World Health Organization Quality of Life-BREF instrument. The WHOQOL-BREF is a 26-item self-report measure that evaluates the quality of life across the domains of physical health (7 points), psychological state (6 points), social relationships (3 points), and environment (8 points) [13-16]. Each item is rated on a 5-point Likert-type scale, providing valuable insights into the individual's overall quality of life over the past two weeks. Domain scores scale in a positive direction (i.e., higher scores mean higher QOL). The score range for each domain is 4-20. The internal consistency of WHOQOLBREF ranged from 0.66 to 0.87 (Cronbach's coefficient). This scale has been proven to be highly discriminatory. These assessments offer a comprehensive understanding of the individual's well-being and can inform personalized interventions and support services to enhance their quality of life. Scores are collected at baseline (pre-intervention) and at the end of the 12-week intervention period (post-intervention) for both the yoga and control groups.

Statistical procedures

The data collected were analyzed using SPSS version 22 for Windows (IBM Corp., Armonk, NY). The normality distribution of the data was checked using the Shapiro-Wilk test; hence, the parametric tests were used. “Independent-sample t-test” and “paired-samples t-test” were used to analyze between and within groups, respectively. The threshold of significance was set at P < 0.05.

## Results

The demographic characteristics of panic disorder patients (n = 64) are summarized in Table 2. The mean age was 25.77 ± 6.01 years, with 54.7% male and 45.3% female participants. Educational details revealed that 98.4% were literate, and the majority had completed high school (29.7%) or graduated (42.2%). The participants were predominantly from semi-urban areas (46.9%), and 54.7% were unmarried. Most were employed (45.3%), and the mean ± SD for the entire sample is presented in Table 2.

**Table 2:** Demographic characteristics of panic disorder patients The sample size (n=64). Mean ± SD, SD: standard deviation.

Variable	n (%)
Age in years (mean ± SD)	25.77 ± 6.01
Gender (%)	
Male	35 (54.7)
Female	29 (45.3)
Educational details	
Illiterate	1 (1.6)
Literate	63 (98.4)
Qualification	
Middle school	9 (14.1)
High School	19 (29.7)
Graduation	27 (42.2)
Post-graduation	8 (12.5)
Professional course	1 (1.6)
Locality	
Urban	15 (23.4)
Semi-urban	30 (46.9)
Rural	19 (29.7)
Marital status	
Married	28 (43.8)
Unmarried	35 (54.7)
Separated/divorced	1 (1.6)
Occupation	
Student	14 (21.9)
Employee	29 (45.3)
Housewife	10 (15.6)
Unemployed	11 (17.2)

Within group comparisons in control group

For the control group (Table 3), pre- and post-intervention comparisons using paired t-tests revealed a significant decrease in HAM-A scores (46.78 ± 4.49 to 31.91 ± 4.66, p = 0.000), with notable effect sizes. The WHOQOL-BREF domains (physical, psychological, social, and environmental) also showed significant improvements post-intervention, with an effect size of 3.25 indicating a large and clinically meaningful change. The strong positive correlation of 0.887 between pre- and post-scores indicates a positive relationship.

**Table 3:** Within control group (pre and post) comparison of HAM-A and WHOQOL-BREF Data are presented as mean ± standard deviation (SD). Correlation coefficients (r) are reported for pre-post comparisons. P-values are considered significant at p < 0.05. Effect sizes are reported as Cohen's d for pre-post comparisons.

Variables	Pre/post	Mean ± SD	Correlation	P-value	Effect size
HAM-A	Pre	46.78 ± 4.49	0.887	0.000	3.25
	Post	31.91 ± 4.66			
Physical domain QOL	Pre	35.04 ± 5.17	0.534	0.002	3.45
	Post	51.67 ± 4.44			
Psychological domain QOL	Pre	26.56 ± 7.39	0.688	0.000	2.75
	Post	46.74 ± 7.31			
Social domain QOL	Pre	10.16 ± 9.87	0.479	0.006	3.64
	Post	45.31 ± 9.45			
Environmental domain QOL	Pre	12.79 ± 8.71	0.681	0.000	3.69
	Post	46.58 ± 9.57			

Within group comparisons in yoga group

In the yoga group (Table 4), significant reductions in HAM-A scores (49.13 ± 4.55 to 13.53 ± 5.54, p<0.001) were observed, indicating a substantial decrease in anxiety levels. The WHOQOL-BREF domains showed significant improvements post-intervention. The correlation of 0.405 suggests a moderate relationship between pre-intervention scores and improvement; those with higher initial anxiety tended to experience greater benefits. The effect size of 7.02 underscores the considerable magnitude of the change, highlighting a substantial clinical impact on anxiety levels.

**Table 4:** Within yoga group (pre and post) comparison of HAM-A and WHOQOL-BREF Data are presented as mean ± standard deviation (SD). Correlation coefficients (r) are reported for pre-post comparisons. P-values are considered significant at p < 0.05 or p < 0.001, as indicated. Effect sizes are reported as Cohen's d for pre-post comparisons.

Variables	Pre/post	Mean ± SD	Correlation	P Value	Effect size
HAM-A	Pre	49.13 ± 4.55	0.405	<0.001	7.02
	Post	13.53 ± 5.54			
Physical domain QOL	Pre	34.82 ± 5.37	0.579	<0.001	4.11
	Post	60.04 ± 6.81			
Psychological domain QOL	Pre	26.17 ± 8.93	0.681	<0.001	4.57
	Post	63.28 ± 7.21			
Social domain QOL	Pre	11.72 ± 13.69	0.626	<0.001	4.13
	Post	67.97 ± 13.57			
Environmental domain QOL	Pre	14.55 ± 14.45	0.597	<0.001	4.51
	Post	72.56 ± 11.05			

Between group comparisons in control versus yoga group

Between-group comparisons (Table 5) highlighted significant differences in HAM-A scores between the control and yoga groups at both pre (p = 0.042) and post (p = 0.000) assessments. Effect sizes suggested a moderate effect at pre-assessment and a large effect at post-assessment. Regarding the quality of life, significant differences favouring the yoga group were found in the physical, psychological, social, and environmental domains post-intervention, with moderate to large effect sizes.

**Table 5:** Between group (control vs yoga) comparison of post HAM-A and WHOQOL-BREF Data are presented as mean ± standard deviation (SD). P-values are considered significant at p < 0.001. Effect sizes are reported as Cohen's d for between-group comparisons.

Variables	Group	Mean ± SD	P-value	Effect size
HAM-A	Control	31.91 ± 4.66	0.000	3.59
	Yoga	13.53 ± 5.54		
Physical domain QOL	Control	51.67 ± 4.44	0.000	1.46
	Yoga	60.04 ± 6.81		
Psychological domain QOL	Control	46.74 ± 7.31	0.000	2.28
	Yoga	63.28 ± 7.21		
Social domain QOL	Control	45.31 ± 9.45	0.000	1.94
	Yoga	67.97 ± 13.57		
Environmental domain QOL	Control	46.58 ± 9.57	0.000	2.51
	Yoga	72.56 ± 11.05		

## Discussion

This article presents a well-designed study investigating the effectiveness of yoga as an intervention for panic disorder. The study provides compelling evidence that yoga can significantly improve anxiety symptoms and quality of life in individuals with panic disorder, potentially offering a complementary or alternative approach to traditional pharmacological and psychotherapeutic treatments. The results demonstrate that there were significant differences in the anxiety and the QOL of the pre-test and the post-tests of the yoga group. These findings align with previous research demonstrating the efficacy of various interventions for treating panic disorder, including CBT and mindfulness-based interventions (MBIs) [17-20].

Consistent with existing literature, the yoga group experienced a significant reduction in anxiety scores, and improvements across all domains of quality of life were observed compared to the control group in the 12th week, highlighting yoga's multifaceted impact on well-being. Large effect sizes further suggest a clinically meaningful benefit of yoga beyond mere statistical significance. This suggests that yoga was effective in alleviating anxiety symptoms and improving the quality of life for individuals with panic disorder.

The study employed a randomized controlled design, considered the gold standard for experimental research. This strengthens the internal validity and allows for causal inferences to be drawn about the effect of yoga on anxiety and quality of life. The study assessed both anxiety symptoms (using the Hamilton Anxiety Rating Scale) and quality of life across various domains (physical, psychological, social, and environmental) using the WHOQOL-BREF instrument. This provides a more holistic understanding of the impact of yoga on individuals' well-being and a nuanced understanding of yoga's influence on both anxiety symptoms and various quality of life aspects. The results demonstrate statistically significant improvements in both anxiety scores and quality of life across all domains within the yoga group compared to the control group. The effect sizes were also large, suggesting a clinically meaningful impact of yoga on the participants. The study employed a standardized yoga programme, outlining the specific yoga practices (asana, pranayama, and meditation) used in the intervention. This allows for replication and comparison with other studies investigating the use of yoga for panic disorder. While the study demonstrates the immediate benefits of yoga, it would be valuable to conduct follow-up assessments to determine if the positive effects are sustained over time. Overall, this study provides strong evidence for the potential of yoga as an effective intervention for panic disorder. The findings encourage further research into the potential benefits of yoga for mental health and well-being and its integration into more comprehensive treatment plans for individuals with anxiety disorders.

Further research is needed to investigate the specific mechanisms by which yoga contributes to the reduction of anxiety and improvement in quality of life. The study used a standardized yoga program. However, individualizing the yoga practice based on each participant's specific needs and preferences might further enhance the therapeutic benefits. The limitation of the study is that the participants were all diagnosed with panic disorder and recruited from a single location. This limits the generalizability of the findings to other populations and settings. Further research with larger and more diverse samples is needed to confirm the generalizability of the findings.

## Conclusions

Yoga effectively reduced anxiety and improved quality of life in panic disorder patients. The yoga group experienced a significantly greater reduction in anxiety scores compared to the control group, and improvements were observed across all domains of quality of life. Future research is needed on long-term benefits, mechanisms of action, and personalization. This study suggests that yoga can be a valuable complementary or alternative approach to traditional treatments for panic disorder.
